# Multiple genetic imaging study of the association between cholesterol metabolism and brain functional alterations in individuals with risk factors for Alzheimer's disease

**DOI:** 10.18632/oncotarget.8100

**Published:** 2016-03-15

**Authors:** Feng Bai, Yonggui Yuan, Yongmei Shi, Zhijun Zhang

**Affiliations:** ^1^ Department of Neurology, Affiliated ZhongDa Hospital, School of Medicine, Southeast University, Nanjing, China

**Keywords:** cholesterol metabolism pathway, imaging genetics, brain function, amnestic mild cognitive impairment, Alzheimer's disease, Gerotarget

## Abstract

Alzheimer's disease (AD) is a clinically and genetically heterogeneous neurodegenerative disease. Genes involved in cholesterol metabolism may play a role in the pathological changes of AD. However, the imaging genetics-based endophenotypes derived from polymorphisms in multiple functionally related genes are unclear in individuals with risk factors for AD. Forty-three amnestic mild cognitive impairment (aMCI) subjects and 30 healthy controls underwent resting-state functional magnetic resonance imaging (fMRI) measurements of brain topological organization. Thirty-three previously suggested tagging single nucleotide polymorphisms (SNPs) from 12 candidate genes in the cholesterol metabolism pathway were further investigated. A cholesterol metabolism pathway gene-based imaging genetics approach was then utilized to investigate disease-related differences between the groups based on genotype-by-aMCI interactions. The cholesterol metabolism pathway genes exerted widespread effects on the cortico-subcortical-cerebellar spontaneous brain activity. Meanwhile, left lateralization of global brain connectivity was associated with cholesterol metabolism pathway genes. The *APOE* rs429358 variation significantly influenced the brain network characteristics, affecting the activation of nodes as well as the connectivity of edges in aMCI subjects. The cholesterol metabolism pathway gene-based imaging genetics approach may provide new opportunities to understand the mechanisms underlying AD and suggested that *APOE* rs429358 is a core genetic variation that is associated with disease-related differences in brain function.

## INTRODUCTION

Alzheimer's disease (AD) is a clinically heterogeneous neurodegenerative disease with a strong genetic component [1]. The characteristic pathological changes identified in AD brain tissue include extracellularly deposited amyloid-β (Aβ) plaques and intracellular neurofibrillary tangles (tau hyperphosphorylation). However, individual susceptibility to this disease remains largely unclear due to the genetic complexity of AD.

Converging evidence from clinical and pathological studies has indicated that genes involved in cholesterol metabolism may have a potential role in the clearance of highly amyloidogenic Aβ from the brain [1, 2]. The underlying mechanism involves the activity of enzymes that are involved in amyloid precursor protein (APP) metabolism and that are influenced by cholesterol. Post-transcriptional processing of APP also occurs in cholesterol-rich membrane domains [3]. In addition, an indirect relationship between cholesterol and tau hyperphosphorylation was observed in a previous study [4]. The role of cholesterol as a causative factor in the progression of AD is still debated. For example, elevated serum and plasma cholesterol levels do not appear to be risk factors for AD [5], and the brain cholesterol level that is associated with AD remains unclear (i.e., reduced cholesterol levels, increased cholesterol levels, and no changes in cholesterol levels have been observed in AD patients compared with controls) [6]. However, a very exciting finding has shown that a set of ten lipids from the peripheral blood can predict phenoconversion to amnestic mild cognitive impairment (aMCI, which is associated with a high risk for AD) or AD within a 2-3 year time frame with over 90% accuracy [7]. Moreover, recent viewpoints support progressive deterioration of cholesterol homeostasis as a central component of AD pathophysiology, and cholesterol homeostasis may be a potential therapeutic target for disease prevention [7, 8]. The cholesterol metabolism hypothesis is a major addition to the amyloid cascade hypothesis and the tau hypothesis underlying the cholesterol-AD interaction and proposes that several different properties and functions of cholesterol are associated with AD [5, 9].

Imaging genetics association studies have provided many new opportunities to understand the neurobiology of the cholesterol metabolism abnormalities that may contribute to AD. Apolipoprotein E (*APOE*) is the most prevalent cholesterol transport protein in the central nervous system [2], whilst it also modifies the brain inflammatory responses [10]. The *APOE* ϵ4 allele is a confirmed genetic risk factor for sporadic AD, while the ϵ2 allele may confer protection against the disease [11]. In a recent study, we demonstrated that the opposing trajectories of the influence of the *APOE* ϵ2 and ϵ4 alleles in the default mode network with age may reflect their antagonistic pleiotropic properties and their association with different AD risk factors [12]. Other genetic polymorphisms associated with the cholesterol metabolism pathway have also been proposed as risk factors that influence the AD brain. For example, Cystatin C (*CST3*) has been suggested to be related to white matter lesions [13], grey matter density [14] and the global neurophysiological phenotype [15] of AD patients and aMCI individuals, and sortilin-related receptor (*SORL1*) gene variants are associated with hippocampal atrophy and white matter hyperintensities in AD patients [16, 17]. However, AD is currently conceptualized as a multifaceted pathology that is also characterized by a high degree of genetic heterogeneity [18], and the relationships between several other cholesterol metabolism genes and neuroimaging are still largely unclear. A single candidate gene-based approach might coherently account for the diversity and lack of independent replications of these associations [19, 20]. Intriguingly, an alternative pathway-based imaging genetics approach has been suggested to address this issue. This approach applies a general linear model with non-stationary cluster-based inference to examine the associations between polymorphisms and brain imaging biomarkers [21]. These findings may strengthen the confidence in image-derived endophenotypes by enabling testing of polymorphisms in multiple functionally related genes.

In this study, our first aim was to test the hypothesis that a cholesterol metabolism genetics pathway-based imaging approach would reveal abnormal topological organization of brain activity in aMCI subjects. The *APOE* gene is the most established genetic risk factor for sporadic AD [1]; therefore, our second aim was to test whether disease-*APOE* single nucleotide polymorphism (SNP) interactions could be used to identify significant functional variations that differentiate aMCI subjects from healthy controls.

## RESULTS

### Participant characteristics

Compared with the controls, the aMCI subjects showed memory impairment as well as deficits on other cognitive assessments, such as the CDR, MMSE, Auditory verbal memory test-delayed recall, Rey-Osterrieth complex figure test-delayed recall, TMT-A, TMT-B, the symbol digit modalities test and the clock-drawing test (Table 1). Namely, the deficits of the participants in the aMCI group were characteristic of aMCI-multiple domain, which reflects impairments in the memory domain plus at least one other cognitive domain. In addition, there was a trend toward lower education levels in the aMCI group (aMCI 13.58 years and controls 14.98 years). There were no significant differences between these groups with regard to age, gender and the digit span test. Genotype frequencies did not deviate from Hardy-Weinberg equilibrium in either group (*P* > 0.05).

**Table 1:** Demographic and neuropsychological data between the aMCI subjects and the controls

Item	aMCI (*n*= 43)	Controls (*n*= 30)	*P*
Age (years)	72.00±4.88	72.93±3.93	0.661†
Education level (years)	13.58±3.10	14.98±2.67	0.045†
Gender (male:female)	27:16	17:13	0.601†
Clinical dementia rating (CDR)	0.5	0	-
Mini mental state exam (MMSE)	27.05±1.53	28.2±1.37	0.002*†
Auditory verbal memory test-delayed recall	−0.64±0.57	0.99±0.62	0.000*
Rey-Osterrieth complex figure test-delayed recall	−0.26±0.96	0.44±0.91	0.003*
Trail making test (TMT)-A	0.25±1.03	−0.37±0.85	0.009*
TMT-B	0.22±1.11	−0.41±0.60	0.006*
Symbol digit modalities test	−0.24±0.94	0.39±0.96	0.007*
Clock drawing test	−0.23±1.14	0.29±0.70	0.032*
Digit span test	−0.13±0.93	0.23±0.99	0.119

Values are the mean ± (SD);

† Notes: P-values were obtained using Mann-Whitney U-tests because the neuropsychological data were not normally distributed. Other P-values were obtained using independent-samples T-tests

* indicates significant differences between groups, P < 0.05.

### Cholesterol metabolism pathway genes

#### (i) Within genes

**Figure 1:**
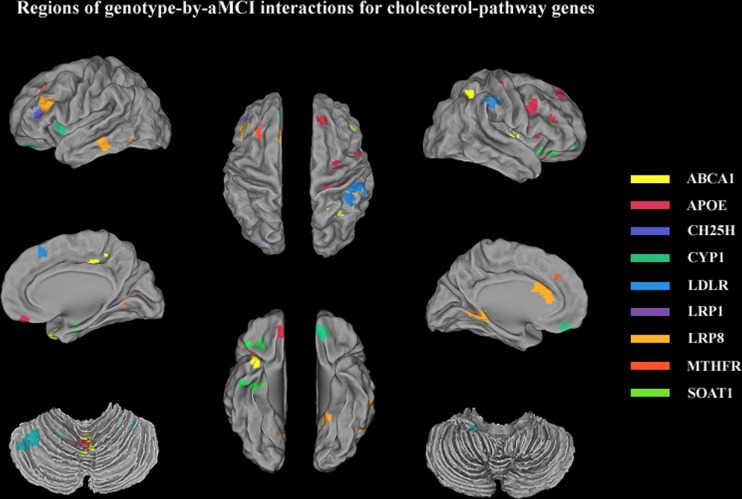
Regions extracted from genotype-by-aMCI interactions for the cholesterol pathway after correcting for the imaging space for any one SNP [*P*^corrected(S)^ < 0.05]: *ABCA1* (rs2230806, yellow); *APOE* (rs7412, rs429358, rs440446, red); *CH25H* (rs4417181, dark blue); *CYP1* (rs754203, rs7157609, green); *LDLR* (rs1433099, rs2738444, blue); *LRP1* (rs1799986, violet); *LRP8* (rs5177, rs3737983, rs3820198, deep yellow); *MTHFR* (rs1801133, orange); and *SOAT1* (rs3753526, grass green) These regions were predominantly components of cortico-subcortical-cerebellar system and included the frontal cortex (superior/medial/middle/inferior frontal gyrus and anterior cingulate), subcortical structures (parahippocampal gyrus and insula), the temporal cortex (superior/middle/inferior temporal gyrus), the parietal cortex (inferior parietal lobule and precuneus), the occipital cortex (middle occipital gyrus), and the cerebellum (posterior lobe/anterior lobe and vermis).

Regions extracted from the genotype-by-aMCI associations that also exhibited spontaneous brain activity (ALFF) differences were identified for 15/33 SNPs of the cholesterol metabolism pathway genes (using a minimum non-stationary AlphaSim-corrected *P*-value for the imaging space for any one SNP [*P*^corrected(S)^ < 0.05)]. These SNPs included *ABCA1* (rs2230806); *APOE* (rs7412, rs429358, rs440446); *CH25H* (rs4417181); *CYP1* (rs754203, rs7157609); *LDLR* (rs1433099, rs2738444); *LRP1* (rs1799986); *LRP8* (rs5177, rs3737983, rs3820198); *MTHFR* (rs1801133); and *SOAT1* (rs3753526). Widespread effects on spontaneous brain activity were predominately observed in components of the cortico-subcortical-cerebellar regions, including the frontal cortex (superior/medial/middle/inferior frontal gyrus and anterior cingulate), subcortical structures (parahippocampal gyrus and insula), the temporal cortex (superior/middle/inferior temporal gyrus), the parietal cortex (inferior parietal lobule and precuneus), the occipital cortex (middle occipital gyrus) and the cerebellum (posterior lobe/anterior lobe and vermis) (for details, see Table 2 and Figure 1). The details of ALFF values in all regions of the ‘SNP-by-status’ interactions for the cholesterol metabolism pathway were shown in the Supplementary Materials (Part II).

**Table 2:** Descriptions of the brain regions extracted from the genotype-by-aMCI interactions that remained statistically significant after correcting for the imaging space and across multiple SNPs per gene

Cholesterol metabolism pathway SNP	Allele	Cluster size	Peak MNI coordinates x, y, z	Peak F value	Brain region	*P*^corrected (S)^ < 0.05	*P*^corrected (S,G)^ < 0.05
***ABCA1* (ATP-binding cassette transporter A1)**							
rs2230806	AG	2214	−21 −102 9	12.7	Middle Occipital Gyrus _L (L.MOG)	*	-
		2241	−15 30 33	9.96	Middle Frontal Gyrus _L (L.MFG)	*	-
		1539	0 −57 0	9.95	Vermis_4_5 (Vermis45)	*	-
***APOE* (Apolipoprotein E)**							
rs7412	CT	2079	−42 27 24	19.15	Inferior Frontal Gyrus_L (L.IFG)	*	-
		2025	−60 −33 −15	15.59	Middle Temporal Gyrus_L (L.MTG)	*	-
		2403	33 −9 15	14.79	Insula_R (R.INS)	*	-
		1809	15 −24 39	14.40	Middle Cingulum Gyrus_R (R. MCG)	*	-
rs405509	AC	-	-	-	None		
rs429358	CT	2349	−36 −57 −33	81.78	Cerebellum Posterior Lobe_L (L. CRBL_Po)	*	**
		1485	36 15 −36	66.04	Superior Temporal Gyrus_R (R.STG)	*	**
		2538	−3 21 18	38.42	Anterior Cingulate Gyrus_L (L.ACG)	*	-
		1998	−12 −45 −3	31.11	Parahippocampal gyrus _L (L.PHG)	*	-
rs440446	CG	2754	15 −48 51	21.37	Precuneus_R (R.PCUN)	*	-
		2133	−21 −51 −36	21.34	Cerebellum Anterior Lobe_L (L.CRBL_Ant)	*	-
		3159	21 −57 −39	17.91	Cerebellum Posterior Lobe_R (R. CRBL_Po)	*	-
		1431	−57 −66 9	15.37	Middle Temporal Gyrus_L (L.MTG)	*	-
rs769450	AG	-	-	-	None		
***CH25H* (Cholesterol 25-Hydroxylase)**							
rs4417181	CT	1809	39 30 15	15.37	Middle Frontal Gyrus_R (R.MFG)	*	-
rs7091822	GT	-	-	-	None		
rs17117126	AG	-	-	-	None		
							
**CST3** (Cystatin C)							
rs2424577	AG	-	-	-	None		
rs3827143	AG				None		
***CYP1* (cytochrome P450 1)**							
rs754203	CT	2916	30 27 −15	17.80	Inferior Frontal Gyrus_R (R.IFG)	*	-
		2079	36 −12 −27	15.94	Parahippocampal gyrus_R (R.PHG)	*	-
rs4900442	CT				None		
rs7157609	AC	2565	30 27 −15	15.00	Inferior Frontal Gyrus_R (R.IFG)	*	-
		2106	33 −12 −27	14.87	Parahippocampal gyrus_R (R.PHG)	*	-
***IDE* (Insulin-degrading enzyme)**							
rs3758505	GT	-	-	-	None		
rs4646954	AG	-	-	-	None		
***LDLR* (Low-density lipoprotein receptor)**							
rs688	CT				None		
rs5925	CT				None		
rs1433099	AG	1296	3 21 48	16.18	Medial Frontal Gyrus_R (R.MeFG)	*	-
rs2738444	CT	5940	45 −36 42	28.40	Inferior Parietal Lobule_R (R.IPL)	*	**
rs11668477	AG	-	-	-	None		
rs12983082	AC	-	-	-	None		
***LRP1* (Low-density lipoprotein receptor-related protein 1)**							
rs1140648	AG	-	-	-	None		
rs1799986	CT	1620	69 −27 −21	16.63	Inferior Temporal Gyrus_R (R.ITG)	*	-
rs2306692	CT	-	-	-	None		
***LRP8* (Low-density lipoprotein receptor-related protein 8)**							
rs5177	CG	3780	48 9 39	27.68	Middle Frontal Gyrus_R (R.MFG)	*	-
		4698	30 24 18	18.91	Insula_R (R.INS)	*	-
		4779	36 −24 54	17.88	Precentral Gyrus_R (R.PreCG)	*	-
		4131	−9 36 −15	16.36	Medial Frontal Gyrus_B (B.MeFG)	*	-
rs3737983	CT	1296	18 36 51	14.93	Superior Frontal Gyrus_R (R.SFG)	*	-
rs3820198	GT	3483	−30 6 3	14.34	Putamen_L (L.Put)	*	-
***MTHFR*(Methylenetetrahydrofolate reductase)**							
rs1801133	CT	1404	−24 −84 15	8.49	Middle Occipital Gyrus_L (L.MOG)	*	-
***PLAU* (Urokinase-plasminogen activator)**							
rs2227564	CT	-	-	-	None		
							
***SOAT1* (Sortilin-related receptor)**							
rs1044925	AC	-	-	-	None		
rs2862616	CT	-	-	-	None		
rs3753526	CG	2916	−39 27 3	22.29	Inferior Frontal Gyrus_L (L.IFG)	*	-

Abbreviations: R= right; L=left; B=Bilateral; Cluster size is in mm^3^; MNI: Montreal Neurological Institute.

* Remained statistically significant after correcting for the imaging space (the statistical threshold was set at *P* < 0.005 and a cluster size of 1296 mm^3^, which corresponds to a corrected *P* < 0.05).

** Remained statistically significant after correcting for the imaging space and across multiple SNPs, *P* < 0.05 [i.e., the original *P*-value was set at *P* < 0.0003 (0.005/15) with a cluster size of 1296 mm^3^].

#### (ii) Network reconstruction and characteristics

**Figure 2:**
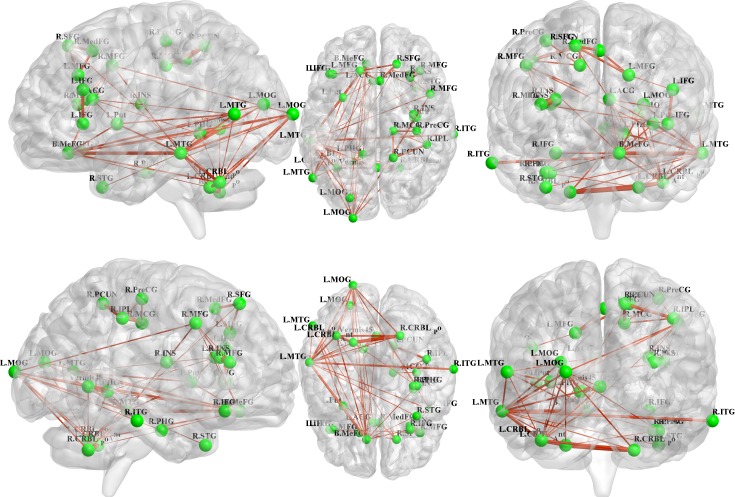
All 31 regions extracted from the genotype-by-aMCI interactions for the cholesterol pathway, after correcting for imaging space for any one SNP [*P*^corrected(S)^ < 0.05], were used to delineate a unidirectional weighted network with 31 nodes and 465 edges that globally described the network connectivity pattern of the control group In the present study, three correlation coefficient thresholds (r = 0.3, r = 0.5 and r = 0.7) were tested. This figure shows the network for r = 0.3, while the networks for r = 0.5 and r = 0.7 are shown in the Supplementary Materials (Part III). The figure was created using BrainNet Viewer (http://www.nitrc.org/projects/bnv/).

**Figure 3:**
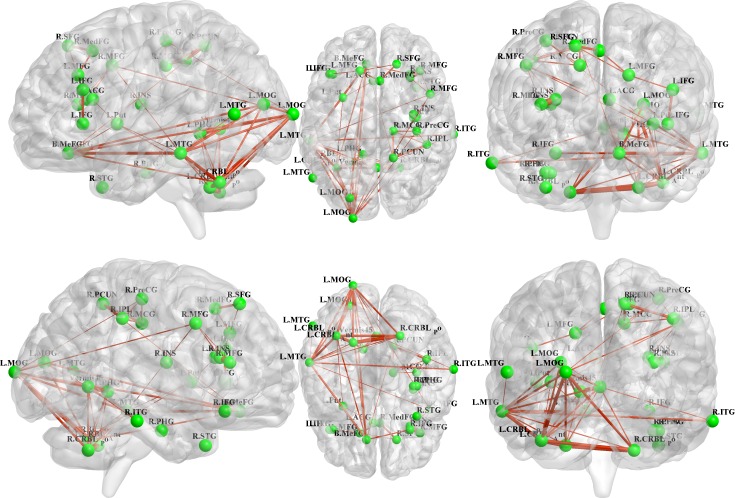
Unidirectional weighted network with 31 nodes and 465 edges for the aMCI group This figure shows the network for r = 0.3, while the patterns for r = 0.5 and r = 0.7 are shown in the Supplementary Materials (Part III). The figure was created using BrainNet Viewer (http://www.nitrc.org/projects/bnv/).

All 31 regions extracted from the genotype-by-aMCI interactions for the cholesterol metabolism pathway genes, after correcting for the imaging space for any one SNP [*P*^corrected(S)^ < 0.05], were used to delineate a unidirectional weighted network with 31 nodes and 465 edges that described the network connectivity patterns. Three different correlation coefficient thresholds (r = 0.3, r = 0.5 and r = 0.7) were used to conduct a comprehensive assessment of network properties. The networks for r = 0.3 are shown in Figure 2 (control group) and Figure 3 (aMCI group), while the networks for r = 0.5 and r = 0.7 are shown in the Supplementary Materials (Part III). A qualitative visual inspection of the connectivity patterns of the reconstructed networks indicated that at corresponding thresholds, the networks for the groups were similar.

**Figure 4:**
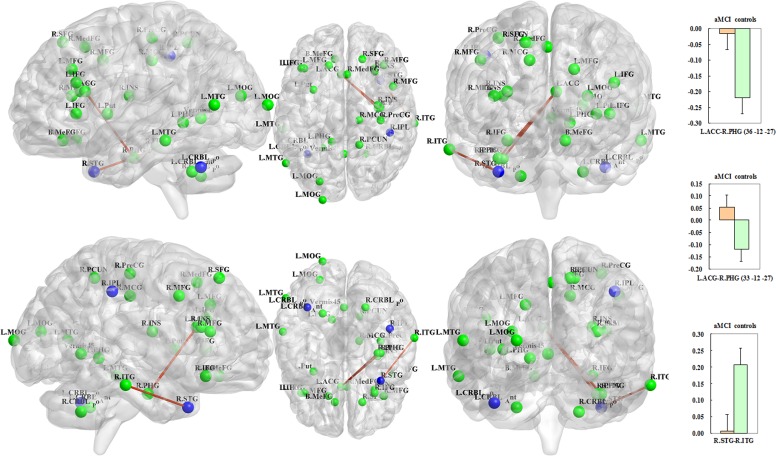
The blue regions remained significant after correcting for the imaging space for any one SNP [*P*^corrected(S)^ < 0.05] and across multiple SNPs per gene [*P*^corrected(S, G)^ < 0.05] and included the left cerebellum posterior lobe (*APOE* rs429358), the right superior temporal gyrus (*APOE* rs429358) and the right inferior parietal lobule (*LDLR* rs2738444) In addition, the three red-brown edges indicate decreased connectivity of the unidirectional weighted network in the aMCI subjects compared to the controls (*P* < 0.005); these edges included the L.ACC (*APOE* rs429358)-R.PHG (*CYP1* rs754203, MNI: 36 −12 −27); L.ACC (*APOE* rs429358)-R.PHG (*CYP1* rs7157609, MNI: 33 −12 −27); and R.STG (*APOE* rs429358)-R.ITG (*LRP1* rs1799986). The correlation coefficient values are shown in the histograms on the right side. The connectivity patterns for the other thresholds (i.e., *P* < 0.01, 0.05 and 0.001) are shown in the Supplementary Materials (Part IV). The figure was created using BrainNet Viewer (http://www.nitrc.org/projects/bnv/).

With respect to the disease-related differences in the neural networks, three edges of the unidirectional weighted network were detected that exhibited decreased connectivity in the aMCI group compared with the control group (*P* < 0.005), including L.ACC (*APOE* rs429358)-R.PHG (*CYP1* rs754203, MNI: 36 −12 −27), L.ACC (*APOE* rs429358)-R.PHG (*CYP1* rs7157609, MNI: 33 −12 −27) and R.STG (*APOE* rs429358)-R.ITG (*LRP1* rs1799986) (Figure 4). Interestingly, all of the differences were associated with *APOE* rs429358. To objectively evaluate these differences, four connectivity thresholds (i.e., *P* < 0.05, 0.01, 0.005 and 0.001) were analysed. The differences with *P* < 0.005 are shown in Figure 4, while the differences identified using the other thresholds of *P* < 0.05, *P* < 0.01 and *P* < 0.001 are shown in the Supplementary Materials (Part IV). The disease-related differences in the neural networks identified with thresholds of *P* < 0.01, 0.005 and 0.001 were similar.

#### (iii) Between genes

Three nodes remained significant between aMCI and controls after correcting for the imaging space for any one SNP [*P*^corrected(S)^ < 0.05] and across multiple SNPs per gene [*P*^corrected(S, G)^ < 0.05]: the left cerebellum posterior lobe, the right superior temporal gyrus and the right inferior parietal lobule (Table 2). Interestingly, 2/3 nodes (i.e., left cerebellum posterior lobe and right superior temporal gyrus) were associated with *APOE* rs429358, while the remaining node (i.e., right inferior parietal lobule) was related to *LDLR* rs2738444 (Table 2; Figure 4).

#### (iv) Gene-brain-behaviour interactions

**Figure 5:**
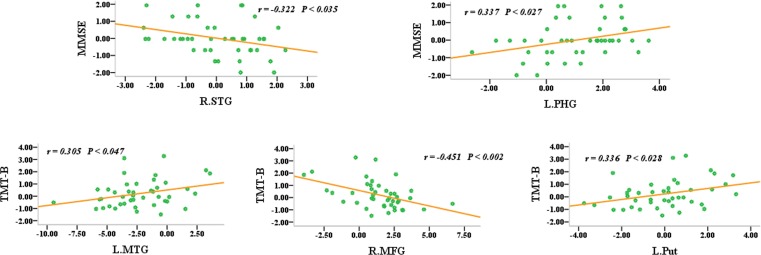
Node strength (i.e., the weighted edges) and their behavioural significance were in the aMCI group The neuropsychological test scores were converted into z-scores.

To evaluate the overall effects of these 31 regions of interest (ROIs) as well as their interactions with aMCI, they were used to delineate a unidirectional weighted network with 31 nodes and 465 edges that described the network connectivity pattern of each participant. The node strength (i.e., weighted edge) and behavioural significance tests were then performed. The MMSE scores were correlated with the node strength of the right superior temporal gyrus (r = 0.322, *P* < 0.035) and the left parahippocampal gyrus (r = 0.337, *P* < 0.027), while the TMT-B scores were correlated with the node strength of the left middle temporal gyrus (r = 0.305, *P* < 0.047), the right middle frontal gyrus (r = −0.451, *P* < 0.002) and the left putamen (r = 0.336, *P* < 0.028) (Figure 5). However, it should be noted that these correlation analysis or partial correlation analysis (age, disease status, education and gender as covariates) did not exhibited significantly statistical robustness after multiple comparison corrections. Therefore, these initial findings should be confirmed in a large sample.

## DISCUSSION

Using genetics pathway-based imaging approaches, this study investigated the topological organization of cholesterol metabolism genes-brain-disease interactions in aMCI subjects. The two main findings were as follows: (i) Cholesterol metabolism pathway genes exerted widespread effects on spontaneous brain activity, especially of regions involving the cortico-subcortical-cerebellar regions. Meanwhile, left lateralization of global brain connectivity was associated with the cholesterol metabolism pathway genes. (ii) The prominent associations of *APOE* gene variations with network characteristics (i.e., nodes and weighted edges) strengthened our hypothesis that polymorphisms linked to the biological functions of *APOE* can be used to distinguish aMCI subjects from healthy controls.

### Combined effect of cholesterol metabolism pathway genes on brain function

Brain cholesterol levels are significantly reduced in the hippocampus and cerebral cortex in AD patients [22]. These brain areas are more likely to exhibit the pathological hallmarks of AD and contain the greatest amounts of synaptic membrane cholesterol, in the following quantitative order: hippocampus > cortex > cerebellum [8, 23]. However, the brain cholesterol levels of AD patients are highly variable [6]. It is indisputable that the brain cholesterol is localized in the specialized myelin and neuronal and glial membranes that are associated with widespread cortical, subcortical and cerebellar areas [9]. Indeed, the progressive deterioration of cholesterol homeostasis in AD plays a central role in the disease's pathophysiology [6]. Accordingly, in the present study, cholesterol metabolism pathway genes exhibited widespread effects on the components of cortico-subcortical-cerebellar regions, and these effects could be related to the underlying pathological mechanisms of the disruption of cholesterol homeostasis. We recently reported gene-brain-behaviour interactions involving tau protein pathway genes and the disrupted topological architecture of cortico-subcortical-cerebellar regions in aMCI subjects [24]. Comparing these two studies, the brain areas identified in the present study were more distributed throughout the cortico-subcortical-cerebellar regions, while the tau protein pathway genes were predominantly associated with subcortical and cerebellar areas. This study is the first to extend our understanding of the detailed differences in neuroimaging patterns that are related to the cholesterol metabolism pathway and the tau protein pathway, although the details remain to be explored in future investigations. In addition, the convergent connectivity in the functional brain network that was constructed from multiple cholesterol metabolism genes showed predominately left lateralization in both the aMCI and control groups. Although the adaptive benefit of the relationship between this specific connectivity and the cholesterol metabolism pathway genes is unclear, the networks associated with this pathway were similar in the control and aMCI subjects. We can assume that these alterations may become more apparent as the disease progresses. Furthermore, the present study demonstrated that the node strength (i.e., the weighted edges) was significantly correlated with cognitive performance in the aMCI group, suggesting that the cholesterol metabolism pathway genes recruited existing connectivity to support the cognitive function of aMCI subjects. These findings are consistent with those of a recent longitudinal population-based study that suggested that a higher rate of cholesterol synthesis was predictive of lower general cognitive performance [25]. Together, the brain imaging findings from the current study add to the evidence in support of targeting the cholesterol pathway as a therapy for disease prevention.

### ***APOE*** rs429358 is a core genetic variation associated with the disease-related characteristics of the neural network

The *APOE* rs429358 variation had a significant effect on the brain network characteristics of the aMCI subjects, influencing the activation of nodes (i.e., the left cerebellum posterior lobe and the right superior temporal gyrus) as well as the connectivity of edges [i.e., L.ACC (*APOE* rs429358)-R.PHG (*CYP1* rs754203, MNI: 36 −12 −27), L.ACC (*APOE* rs429358)-R.PHG (*CYP1* rs7157609, MNI: 33 −12 −27) and R.STG (*APOE* rs429358)-R.ITG (*LRP1* rs1799986)]. This is consistent with previous studies that showed that the parahippocampal gyrus [26, 27], temporal cortex [28] and cerebellum posterior lobe [29] exhibit substantial structural and functional changes in AD and aMCI subjects. However, the present findings further highlighted the influence of the *APOE* rs429358 variation on cholesterol metabolism pathway genes in individuals at risk for AD. Two *APOE* SNPs, rs429358 and rs7412, facilitated the identification of different *APOE* alleles. More importantly, rs429358 is the SNP that makes the difference between *APOE* ϵ4 or *APOE* ϵ3/2. Therefore, this study suggests that *APOE* may inherently affect the disease-related differentiation of brain function. *APOE* is the most prevalent lipoprotein in the central nervous system [2] and is the major known genetic risk factor for late-onset AD [30]. Recent evidence has shown that *APOE* coordinates the mobilization and redistribution of cholesterol in the repair, growth and maintenance of myelin and neuronal membranes [8], and it is thought to be instrumental in establishing healthy communication connections between neurons in different brain regions [31]. Genetic neuroimaging studies further support the antagonistic pleiotropy hypothesis, which indicates that the *APOE* ϵ4 allele confers some cognitive advantage in early life, despite its adverse consequences in old age [32]. In a recent study, we demonstrated that the association of the *APOE* ϵ2 and ϵ4 alleles with the default mode network changed in opposite directions with age [12]. Moreover, the *APOE* ϵ4 allele is associated with modified brain function and structure in AD patients [33, 34]. In the present study, the *APOE* SNP rs429358 was a core genetic variation that was also associated with network characteristics, and strengthened our hypothesis that polymorphisms linked to the biological functions of *APOE* can be used to distinguish aMCI subjects from healthy controls. These results suggest that the evidence provided by pathway-based imaging approaches may help identify additional genetic biomarkers of individuals at risk for AD.

### Methodological issues

This study has several limitations. First, AD is considered to have a high degree of genetic heterogeneity [18], and the consideration of more complex pathways and genes as well as large-scale sequencing projects are necessary and important to comprehensively describe the patterns of brain changes in patients. Second, it was cross-sectional and some of the aMCI subjects may not have displayed the underlying AD pathology, so it remains to be seen whether the brain functional alterations here in aMCI are related to a progression to AD in the follow-up study. Third, to explore underlying mechanism of the functional alterations, a complementary methodology may be required to support the current results, i.e., DTI connectivity analysis could quantify structural integrity of these proposed pathways. Fourth, the SNP effect on the endophenotypes controlled by disease status is indeed an alternative approach. The advantage of this method is to isolate the genetic effect component which cannot be attributed to disease-status difference and is thus likely due to genuine genetic differences. Therefore, this method may be as a supplement of the present the ‘SNP-by-status’ effect. Finally, ROIs were associated with SNP-by-status effect on ALFF and then to see connectivity changes between controls and aMCI between these identified regions, which may produce false positive results. However, ALFF itself can't explain whether an unusual brain functional circuit and where the specific abnormalities are in the same dataset. To completely avoid circular reasoning [35], future study should split the data in half base on a large sample size: carrying on the fMRI example, ROIs are selected with one half of the data, and then the remainder is employed for statistical analysis within those selected regions. Therefore, these present findings should be interpreted with caution.

In summary, the identification of gene variants may provide new opportunities to understand the mechanisms underlying AD and could contribute to the diagnosis and targeted treatment of individuals at risk for AD. Accordingly, the results of our pathway-based imaging genetics analysis revealed that cholesterol metabolism pathway genes have a widespread effect on spontaneous brain activity, and *APOE* rs429358 is a core genetic variation that is associated with the disease-related differentiation of brain function.

## MATERIALS AND METHODS

### Subjects

The methods were performed in accordance with approved guidelines. The Research Ethics Committee of the Affiliated Zhong-Da Hospital of Southeast University approved the experimental protocols, and informed consent was obtained from all subjects. Briefly, 43 aMCI subjects and 30 healthy controls were recruited. The recruitment of aMCI subjects included those with single-domain aMCI (only memory impairment) and multiple-domain aMCI (memory impairment plus at least one other cognitive domain). All aMCI subjects were included in the study according to the recommendations of Petersen et al. (1999) [36] and others [37]: (i) subjective memory impairment corroborated by the subject and an informant; (ii) objective memory performance documented by an AVLT-delayed recall score less than or equal to 1.5 SD of age- and education-adjusted norms (cut-off of ≤ 4 correct responses on 12 items for patients with ≥ 8 years of education); (iii) MMSE score of 24 or higher; (iv) CDR of 0.5; (v) no or minimal impairment in daily activities; and (vi) absence of dementia or insufficient dementia to meet the NINCDS-ADRDA (National Institute of Neurological and Communicative Disorders and Stroke and the Alzheimer's Disease and Related Disorders Association) Alzheimer's Criteria. In addition, a CDR of 0, an MMSE score ≥ 26, and an AVLT-delayed recall score > 4 were required for control subjects with 8 or more years of education.

### Exclusion criteria

Participants with a known history of stroke, alcoholism, head injury, Parkinson's disease, epilepsy, major depression or other neurological or psychiatric illness, major medical illness, or severe visual or hearing loss were not included in this study.

### SNP genotyping and selection of candidate cholesterol metabolism pathway genes

Genotype analysis was performed by investigators who were blinded to all subjects. First, blood samples were obtained from the 73 subjects. The data were processed and analysed as in our previous study [20]. In detail, DNA was extracted with the TIANamp genomic DNA kit. Then, genotyping was performed using the iPLEX Assay (SEQUENOM iPLEXH Gold Reagent Kit), which involved assay design, DNA isolation, PCR amplification, SAP treatment, adjustment of the extension primers, iPLEX reaction, resin cleaning, dispensing to the SpectroCHIP bioarray, and matrix-assisted laser desorption ionization time-of-flight mass spectrometry (MALDI-TOF MS) analysis. Based on the AD cholesterol metabolism hypothesis [20], 33 previously suggested tagging SNPs from 12 candidate genes were selected: *ABCA1*, *APOE*, *CH25H*, *CST3*, *CYP1*, *IDE*, *LDLR*, *LRP1*, *LRP8*, *MTHFR*, *PLAU* and *SOAT1* (for details, see Results section). Hardy-Weinberg equilibrium was used with χ^2^ tests. SNPs were excluded from the study if (i) they demonstrated complete linkage disequilibrium (LD) with another SNP (i.e., the alleles were completely correlated with the alleles of another SNP); or (ii) the minor allele frequency (MAF) was lower than 5% [21, 24]. Consequently, 33 SNPs from 12 candidate genes could be detected in all subjects and were entered into the following analysis (for a flow sheet, see Part I of Supplemental Materials)

### MRI data acquisition

A General Electric 1.5-Tesla scanner (General Electric Medical Systems, USA) with a homogeneous birdcage head coil was used in this study. First, conventional axial Fast Relaxation Fast Spin Echo sequence T2-weighted anatomical MR images were obtained to rule out major white matter changes, cerebral infarction or other lesions using the following parameters: repetition time (TR) = 3500 ms; echo time (TE) = 103 ms; flip angle (FA) = 90°; acquisition matrix = 320 × 192; field of view (FOV) = 240 mm × 240 mm; thickness = 6.0 mm; gap = 0 mm; and no. of excitations (NEX) = 2.0. Second, high-resolution, T1-weighted axial images covering the whole brain were acquired using a 3D spoiled gradient echo sequence as follows: TR = 9.9 ms; TE = 2.1 ms; FA = 15°; acquisition matrix = 256×192; FOV = 240 mm × 240 mm; thickness = 2.0 mm; and gap = 0 mm. Finally, the functional scans (T2*-weighted images) involved the acquisition of 30 contiguous axial slices using a GRE-EPI pulse sequence: TR = 3000 ms; TE = 40 ms; FA = 90°; acquisition matrix = 64 × 64; FOV = 240mm × 240 mm; thickness = 4.0 mm; gap = 0 mm and 3.75 × 3.75 mm^2^ in-plane resolution parallel to the anterior commissure-posterior commissure line. In all, 142 functional volumes were generated in 7 min and 6 s.

### Data preprocessing

Data analyses were performed with SPM5 software (http://www.fil.ion.ucl.ac.uk/spm). The first eight volumes of the scanning session were discarded to allow for T1 equilibration effects. The remaining images were corrected for timing differences and motion effects. Participants with head motion of more than 3 mm maximum displacement in any direction, x, y, or z, or 3 degrees of any type of angular motion were excluded. Then, the resulting images were spatially normalized into the SPM5 Montreal Neurological Institute echo-planar imaging template using the default settings and resampling to 3 × 3 ×3 mm^3^ voxels. The normalized images were smoothed with a Gaussian kernel of 8 × 8 × 8 mm.

### ALFF analysis

REST software (http://www.restingfmri.sourceforge.net) was used to remove the linear trends of time courses and for temporal band-pass filtering (0.01-0.08 Hz). Separate ALFF analyses were then performed for the data of all subjects using this software. Briefly, the time series of the resulting images was transformed to the frequency domain after image preprocessing using a fast Fourier transform (FFT; taper percentage = 0, FFT length = shortest), and the power spectrum was then obtained. Because spectral power is the square of spectral amplitude, the square root was calculated at each frequency of the power spectrum and averaged between 0.01-0.08 Hz for each voxel. The activity in this frequency band was then taken as the ALFF. Only regions within the brain were considered, and the background and other tissues outside the brain were removed.

### Voxelwise-based grey matter volume correction

To control for possible structural differences in the brain ALFF results, we conducted voxelwise-based grey matter volume correction. This correction method includes a voxel's likelihood of containing grey matter as a covariate (nuisance variable) in the analysis of the functional data using standard statistical techniques [24, 38]. The purpose of this method is to isolate the components of the functional changes that cannot be attributed to anatomical differences and are, therefore, likely due to genuine functional differences. In detail, we first used voxel-based morphometry (VBM) to explore the grey matter volume maps of every subject. These maps were transformed into the same standard space as the resting-state fMRI images using offline linear registration. Because VBM results can be sensitive to the size of the smoothing kernel used to smooth the tissue segment images, the criterion applied here was to match the smoothness of the grey matter volume map data to that of the corresponding functional data (8 mm). Finally, the resulting voxelwise grey matter volume maps were input as covariates in the analysis of the functional data. The voxelwise-based grey matter volume correction was applied to the data of each subject. There were no anatomical images for one aMCI participant.

### Statistical analysis

#### (i) Mass univariate modelling

The overall procedure was similar to that of previous studies [21, 24, 39]. Briefly, a general linear model was used to analyse genotype-by-aMCI interactions using the ALFF data at each SNP. Accordingly, in the present study, 33 group × genotype ANOVAs were performed separately (groups: aMCI and controls; genotypes: 2- or 3-level covariate for genotype status). To address the SNPs of the 10% of participants who showed a rare genotype [i.e., $\text{MAF }>\;\sqrt{0.10}\left(\text{Equation }1\right)=\;0.31$], we used a genotypic model that was parameterised with orthogonal polynomials and 2 degrees of freedom. For SNPs with MAF < 0.31, a recessive model merged the rare homozygous and heterozygous groups. In details, genotypes and disease status were the variables of interests, while age, gender and education were set as confounding variables that may be explained by brain functional differences in the samples during the ANOVA. All ANOVA statistical thresholds were set at an AlphaSim-corrected *P* < 0.05 as determined by Monte Carlo simulation (single voxel *P*-value = 0.005, a minimum cluster size of 1296 mm^3^, and FWHM = 8 mm with mask).

#### (ii) Cluster-based inference

Non-stationary cluster-size inference [21, 24, 39] has been used to test for associations between individual SNPs and brain variations while correcting for searching across the entire brain imaging space by using a minimum non-stationary AlphaSim-corrected *P*-value as a summary measure to reflect the ALFF of each SNP within each gene [denoted as *P*^corrected(S)^ < 0.05]. For cases in which multiple SNPs related to the same gene (i.e., *APOE* gene of the present study included rs7412, rs405509, rs429358, rs440446 and rs769450) could be tested, we selected the SNPs that had the statistical threshold with *P*^corrected(S)^ < 0.05 (i.e., rs7412, rs429358 and rs440446 were survive at this threshold) for further analysis after applying a Bonferroni correction based on the number of SNPs within this gene. Thus, the final measure of significance for each gene was corrected for multiple comparisons both within [i.e., the SNPs that had the statistical threshold with *P*^corrected(S)^ < 0.05 in each gene] and between genes [i.e., 15 SNPs of all 12 genes were survive at the threshold of *P*^corrected(S)^ < 0.05, thus the original *P*-value should be set at *P* < 0.005/15, details see Table 2] [denoted as *P*^corrected(S, G)^ < 0.05].

#### (iii) Network reconstruction and behavioural significance

The genotype-by-aMCI associations with ALFF differences remained significant after correcting for the imaging space for any one SNP [*P*^corrected(S)^ < 0.05], including *ABCA1* (rs2230806), *APOE* (rs7412, rs429358, rs440446), *CH25H* (rs4417181), *CYP1* (rs754203, rs7157609), *LDLR* (rs1433099, rs2738444), *LRP1* (rs1799986), *LRP8* (rs5177, rs3737983, rs3820198), *MTHFR* (rs1801133) and *SOAT1* (rs3753526) (also see Results section). Together, 31 clusters associated with the aforementioned SNPs were extracted as ROIs (also see Results section). They were visualized with BrainNet Viewer [40]. To evaluate the overall effects of these 31 ROIs in the aMCI subjects and the controls, these ROIs were used to delineate a unidirectional weighted network. Then, the topological characteristics of the network created by merging the regions described above were examined using the following steps: (i) Network node definitions, where 31 ROIs were extracted from all genotype-by-aMCI interactions [*P*^corrected(S)^ < 0.05]. (ii) Network edge definitions were used to individually extract the averaged BOLD time from the 31 ROIs for each participant. A Pearson's correlation coefficient (after Fisher's z-transform) was obtained for each of the 31 ROI-time series pairs. Thus, we obtained a 31 × 31 matrix for each participant, and the weight of the edge between any two nodes represented the z-value strength of the functional connectivity between the two corresponding brain regions. (iii) All 31 ROIs extracted from the genotype-by-aMCI interactions for the cholesterol metabolism pathway, after correcting for the imaging space for any one SNP [*P*^corrected(S)^ < 0.05], were used to delineate a unidirectional weighted network with 31 nodes and 465 edges that globally described the network connectivity patterns of each participant. *S_i_* quantifies the extent to which a node is relevant to the network and is defined as follows:

$$\text{Si}=\sum_{i}\text{wij}$$ (Equation 2)

where *w_ij_* denotes the weighted edge that connects node *i* and node *j*; in other words, it is the z-value strength of the functional connectivity between brain region *i* and brain region *j*. (iv) Finally, Pearson's correlational analyses between *S_i_* and cognitive performance were performed (*P* < 0.05).

## SUPPLEMENTARY MATERIAL



## References

[1] Karch CM, Cruchaga C, Goate AM (2014). Alzheimer's disease genetics: from the bench to the clinic. Neuron.

[2] Wollmer MA (2010). Cholesterol-related genes in Alzheimer's disease. Biochim Biophys Acta.

[3] Shobab LA, Hsiung GY, Feldman HH (2005). Cholesterol in Alzheimer's disease. Lancet Neurol.

[4] Rahman A, Akterin S, Flores-Morales A, Crisby M, Kivipelto M, Schultzberg M, Cedazo-Mínguez A (2005). High cholesterol diet induces tau hyperphosphorylation in apolipoprotein E deficient mice. FEBS Lett.

[5] Wood WG, Li L, Müller WE, Eckert GP (2014). Cholesterol as a causative factor in Alzheimer's disease: a debatable hypothesis. J Neurochem.

[6] Wood WG, Igbavboa U, Eckert GP, Johnson-Anuna LN, Müller WE (2005). Is hypercholesterolemia a risk factor for Alzheimer's disease?. Mol Neurobiol.

[7] Mapstone M, Cheema AK, Fiandaca MS, Zhong X, Mhyre TR, MacArthur LH, Hall WJ, Fisher SG, Peterson DR, Haley JM, Nazar MD, Rich SA, Berlau DJ (2014). Plasma phospholipids identify antecedent memory impairment in older adults. Nat Med.

[8] Leduc V, Jasmin-Bélanger S, Poirier J (2010). APOE and cholesterol homeostasis in Alzheimer's disease. Trends Mol Med.

[9] Mathew A, Yoshida Y, Maekawa T, Kumar DS (2011). Alzheimer's disease: cholesterol a menace?. Brain Res Bull.

[10] Laskowitz DT, Vitek MP (2007). Apolipoprotein E and neurological disease: therapeutic potential and pharmacogenomic interactions. Pharmacogenomics.

[11] Trachtenberg AJ, Filippini N, Cheeseman J, Duff EP, Neville MJ, Ebmeier KP, Karpe F, Mackay CE (2012). The effects of APOE on brain activity do not simply reflect the risk of Alzheimer's disease. Neurobiol Aging.

[12] Shu H, Shi Y, Chen G, Wang Z, Liu D, Yue C, Ward BD, Li W, Xu Z, Chen G, Guo Q, Xu J, Li SJ (2016). Opposite Neural Trajectories of Apolipoprotein E ϵ4 and ϵ2 Alleles with Aging Associated with Different Risks of Alzheimer's Disease. Cereb Cortex.

[13] Galluzzi S, Geroldi C, Benussi L, Ghidoni R, Testa C, Borsci G, Bonetti M, Manfellotto D, Romanelli G, Zulli R, Binetti G, Frisoni GB (2008). Association of blood pressure and genetic background with white matter lesions in patients with mild cognitive impairment. J Gerontol A Biol Sci Med Sci.

[14] Sloan CD, Shen L, West JD, Wishart HA, Flashman LA, Rabin LA, Santulli RB, Guerin SJ, Rhodes CH, Tsongalis GJ, McAllister TW, Ahles TA, Lee SL (2010). Genetic pathway-based hierarchical clustering analysis of older adults with cognitive complaints and amnestic mild cognitive impairment using clinical and neuroimaging phenotypes. Am J Med Genet B Neuropsychiatr Genet.

[15] Babiloni C, Benussi L, Binetti G, Bosco P, Busonero G, Cesaretti S, Dal Forno G, Del Percio C, Ferri R, Frisoni G, Ghidoni R, Rodriguez G, Squitti R (2006). Genotype (cystatin C) and EEG phenotype in Alzheimer disease and mild cognitive impairment: a multicentric study. Neuroimage.

[16] Assareh AA, Piguet O, Lye TC, Mather KA, Broe GA, Schofield PR, Sachdev PS, Kwok JB (2014). Association of SORL1 gene variants with hippocampal and cerebral atrophy and Alzheimer's disease. Curr Alzheimer Res.

[17] Reitz C, Cheng R, Rogaeva E, Lee JH, Tokuhiro S, Zou F, Bettens K, Sleegers K, Tan EK, Kimura R, Shibata N, Arai H, Kamboh MI (2011). Meta-analysis of the association between variants in SORL1 and Alzheimer disease. Arch Neurol.

[18] Hampel H, Lista S, Teipel SJ, Garaci F, Nisticò R, Blennow K, Zetterberg H, Bertram L, Duyckaerts C, Bakardjian H, Drzezga A, Colliot O, Epelbaum S (2014). Perspective on future role of biological markers in clinical therapy trials of Alzheimer's disease: a long-range point of view beyond 2020. Biochem Pharmacol.

[19] Williamson J, Goldman J, Marder KS (2009). Genetic aspects of Alzheimer disease. Neurologist.

[20] Liu X, Yue C, Xu Z, Shu H, Pu M, Yu H, Shi Y, Zhuang L, Xu X, Zhang Z (2012). Association study of candidate gene polymorphisms with amnestic mild cognitive impairment in a Chinese population. PLoS One.

[21] Inkster B, Nichols TE, Saemann PG, Auer DP, Holsboer F, Muglia P, Matthews PM (2010). Pathway-based approaches to imaging genetics association studies: Wnt signaling, GSK3beta substrates and major depression. Neuroimage.

[22] Mulder M, Ravid R, Swaab DF, de Kloet ER, Haasdijk ED, Julk J, van der Boom JJ, Havekes LM (1998). Reduced levels of cholesterol, phospholipids, and fatty acids in cerebrospinal fluid of Alzheimer disease patients are not related to apolipoprotein E4. Alzheimer Dis Assoc Disord.

[23] Chochina SV, Avdulov NA, Igbavboa U, Cleary JP, O'Hare EO, Wood WG (2001). Amyloid beta-peptide1-40 increases neuronal membrane fluidity: role of cholesterol and brain region. J Lipid Res.

[24] Bai F, Liao W, Yue C, Pu M, Shi Y, Yu H, Yuan Y, Geng L, Zhang Z (2016). Genetics pathway-based imaging approaches in Chinese Han population with Alzheimer's disease risk.

[25] van den Kommer TN, Dik MG, Comijs HC, Lütjohann D, Lips P, Jonker C, Deeg DJ (2012). The role of extracerebral cholesterol homeostasis and ApoE e4 in cognitive decline. Neurobiol Aging.

[26] Echávarri C, Aalten P, Uylings HB, Jacobs HI, Visser PJ, Gronenschild EH, Verhey FR, Burgmans S (2011). Atrophy in the parahippocampal gyrus as an early biomarker of Alzheimer's disease. Brain Struct Funct.

[27] Jacobs HI, Wiese S, van de Ven V, Gronenschild EH, Verhey FR, Matthews PM (2015). Relevance of parahippocampal-locus coeruleus connectivity to memory in early dementia. Neurobiol Aging.

[28] Bai F, Zhang Z, Watson DR, Yu H, Shi Y, Yuan Y, Zang Y, Zhu C, Qian Y (2009). Abnormal functional connectivity of hippocampus during episodic memory retrieval processing network in amnestic mild cognitive impairment. Biol Psychiatry.

[29] Bai F, Liao W, Watson DR, Shi Y, Yuan Y, Cohen AD, Xie C, Wang Y, Yue C, Teng Y, Wu D, Jia J, Zhang Z (2011). Mapping the altered patterns of cerebellar resting-state function in longitudinal amnestic mild cognitive impairment patients. J Alzheimers Dis.

[30] Wolf AB, Caselli RJ, Reiman EM, Valla J (2013). APOE and neuroenergetics: an emerging paradigm in Alzheimer's disease. Neurobiol Aging.

[31] Trachtenberg AJ, Filippini N, Ebmeier KP, Smith SM, Karpe F, Mackay CE (2012). The effects of APOE on the functional architecture of the resting brain. Neuroimage.

[32] Rusted JM, Evans SL, King SL, Dowell N, Tabet N, Tofts PS (2013). APOE e4 polymorphism in young adults is associated with improved attention and indexed by distinct neural signatures. Neuroimage.

[33] Cosentino S, Scarmeas N, Helzner E, Glymour MM, Brandt J, Albert M, Blacker D, Stern Y (2008). APOE epsilon 4 allele predicts faster cognitive decline in mild Alzheimer disease. Neurology.

[34] Filippini N, Rao A, Wetten S, Gibson RA, Borrie M, Guzman D, Kertesz A, Loy-English I, Williams J, Nichols T, Whitcher B, Matthews PM (2009). Anatomically-distinct genetic associations of APOE epsilon4 allele load with regional cortical atrophy in Alzheimer's disease. Neuroimage.

[35] Kriegeskorte N, Simmons WK, Bellgowan PS, Baker CI (2009). Circular analysis in systems neuroscience: the dangers of double dipping. Nat Neurosci.

[36] Petersen RC, Negash S (2008). Mild cognitive impairment: an overview. CNS Spectr.

[37] Winblad B, Palmer K, Kivipelto M, Jelic V, Fratiglioni L, Wahlund LO, Nordberg A, Bäckman L, Albert M, Almkvist O, Arai H, Basun H, Blennow K (2004). Mild cognitive impairment--beyond controversies, towards a consensus: report of the International Working Group on Mild Cognitive Impairment. J Intern Med.

[38] Oakes TR, Fox AS, Johnstone T, Chung MK, Kalin N, Davidson RJ (2007). Integrating VBM into the General Linear Model with voxelwise anatomical covariates. Neuroimage.

[39] Inkster B, Nichols TE, Saemann PG, Auer DP, Holsboer F, Muglia P, Matthews PM (2009). Association of GSK3beta polymorphisms with brain structural changes in major depressive disorder. Arch Gen Psychiatry.

[40] Xia M, Wang J, He Y (2013). BrainNet Viewer: A Network Visualization Tool for Human Brain Connectomics. PLoS One.

