# Supplementary material for: Multiple genetic imaging study of the association between cholesterol metabolism and brain functional alterations in individuals with risk factors for Alzheimer's disease
Source: Oncotarget. 2016 Mar 15;7(13):15315–28. doi: 10.18632/oncotarget.8100 (PMC4941243; doi:10.18632/oncotarget.8100)

Multiple genetic imaging study of the association between cholesterol metabolism and brain functional alterations in individuals with risk factors for Alzheimer's disease

Supplementary Material

Part I. Overview of data process

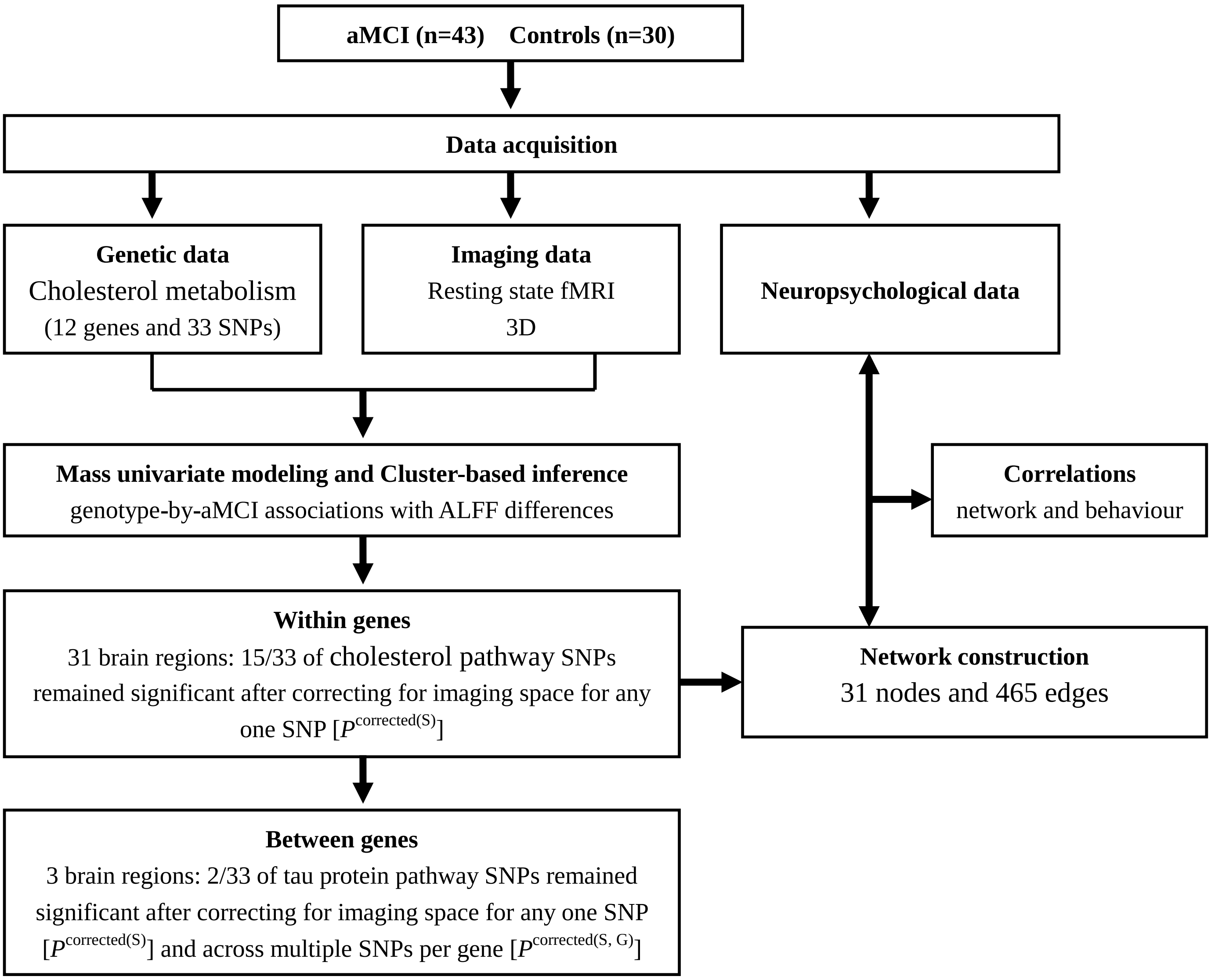

Part II. The details of mean ALFF values in all 31 regions of the 'SNP-by-status' interactions for the cholesterol metabolism pathway after correcting for imaging space for any one SNP ($P^{corrected(S)} < 0.05$), respectively.

ALFF values in all 31 regions of the 'SNP-by-status' interactions

Number	SNPs	Region	ALFF values		*P*
			Controls	aMCI	
	**ABCA1**				
1	rs2230806	L.MOG	2.19±0.47	2.20±0.41	0.413
2		L.MFG	0.88±0.09	0.91±0.10	0.324
3		Vermis45	0.94±0.18	0.99±0.33	0.552
	**APOE**				
4	rs7412	L.IFG	0.99±0.12	0.95±0.09	0.178
5		L.MTG	1.06±0.14	0.96±0.15	0.004*
6		R.INS	0.94±0.09	0.91±0.09	0.145
7		R. MCG	0.86±0.07	0.85±0.08	0.473
8	rs429358	L.CRBL_Po	1.07±0.25	1.06±0.20	0.598
9		R.STG	1.04±0.33	1.05±0.24	0.382
10		L.ACG	1.10±0.22	1.10±0.16	0.523
11		L.PHG	1.05±0.16	1.06±0.12	0.614
12	rs440446	R.PCUN	0.86±0.09	0.84±0.07	0.193
13		L.CRBL_Ant	0.75±0.09	0.78±0.12	0.239
14		R. CRBL_Po	0.72±0.08	0.72±0.09	0.955
15		L.MTG	1.10±0.15	1.07±0.18	0.388
	**CH25H**				
16	rs4417181	R.MFG	0.95±0.09	0.92±0.08	0.433
	**CYP1**				
17	rs754203	R.IFG	0.90±0.17	0.85±0.12	0.193
18		R.PHG	0.76±0.13	0.71±0.08	0.050*
19	rs7157609	R.IFG	0.90±0.17	0.83±0.13	0.088
20		R.PHG	0.87±0.13	0.81±0.10	0.045*
	**LDLR**				
21	rs1433099	R.MeFG	0.85±0.13	0.85±0.12	0.805
22	rs2738444	R.IPL	0.87±0.13	0.85±0.07	0.964

	**LRP1**				
23	rs1799986	R.ITG	0.63±0.11	0.63±0.10	0.920
	**LRP8**				
24	rs5177	R.MFG	0.93±0.08	0.93±0.10	0.911
25		R.INS	0.92±0.08	0.93±0.10	0.480
26		R.PreCG	0.84±0.07	0.81±0.07	0.137
27		B.MeFG	1.14±0.20	1.20±0.26	0.902
28	rs3737983	R.SFG	0.82±0.14	0.81±0.12	0.991
29	rs3820198	L.Put	0.95±0.11	0.90±0.09	0.040*
	**MTHFR**				
30	rs1801133	L.MOG	1.23±0.33	1.20±0.39	0.358
	**SOAT1**				
31	rs3753526	L.IFG	1.13±0.15	1.13±0.20	0.695

Values are the mean ± (SD); Notes: *P* value was obtained by the Mann-Whitney U-test. * indicates significant differences between groups, $P < 0.05$.

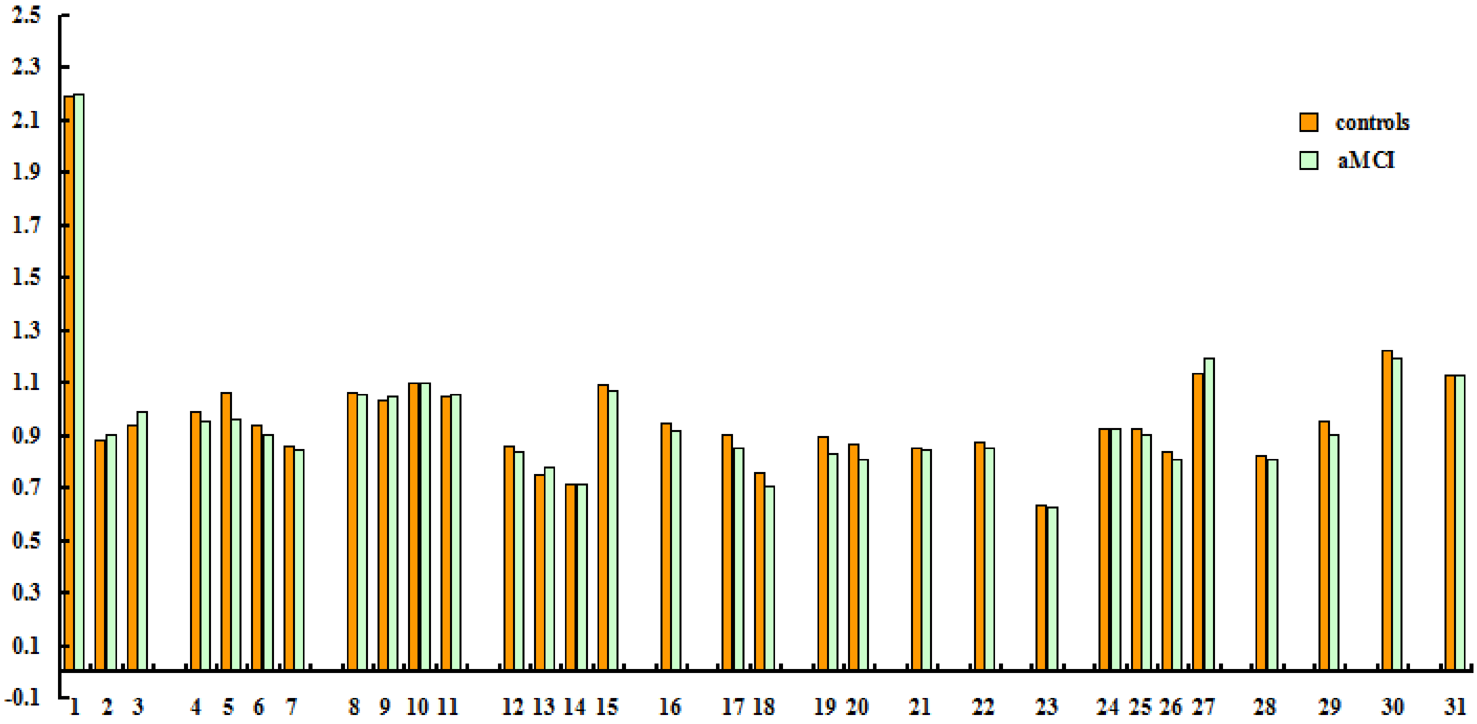

Part III. All 31 regions of genotype-by-aMCI interactions for cholesterol metabolism pathway after correcting for imaging space for any one SNP ($P^{corrected(S)} < 0.05$), which were marked out a unidirectional weighted network with 31 nodes and

465 edges that globally described the network connectivity patterns. The followings are the patterns of r = 0.5 and r = 0.7 in controls and aMCI, respectively.

(1) Controls group r = 0.5

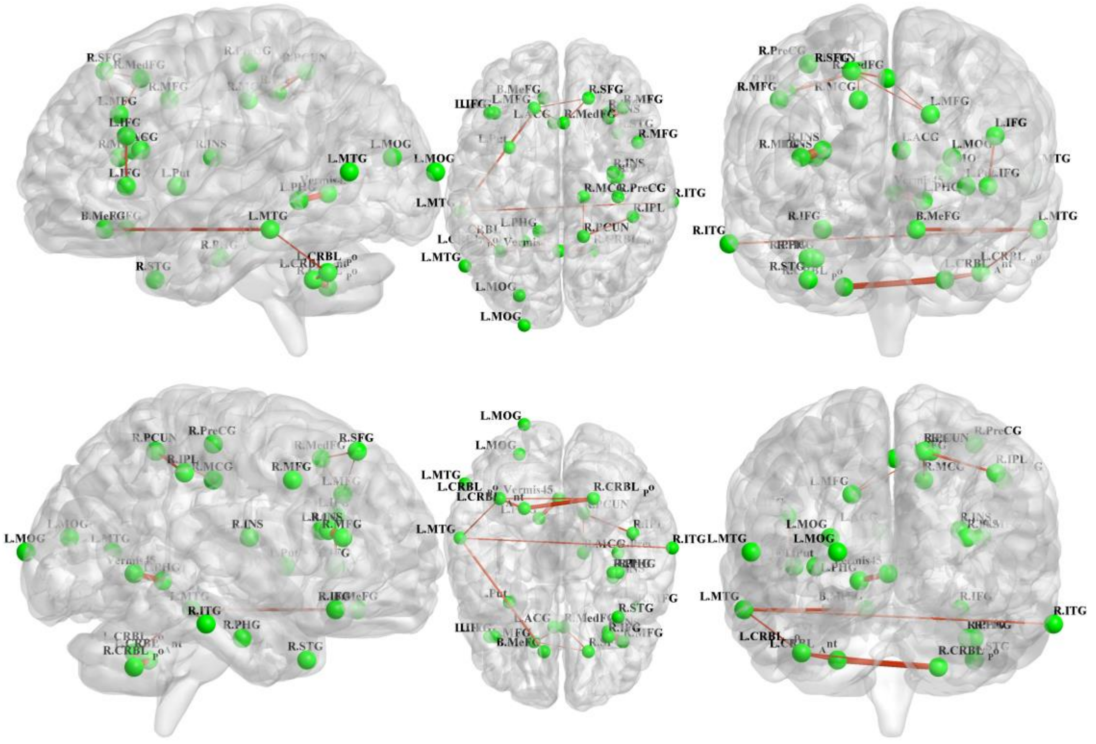

(2) Controls group r = 0.7

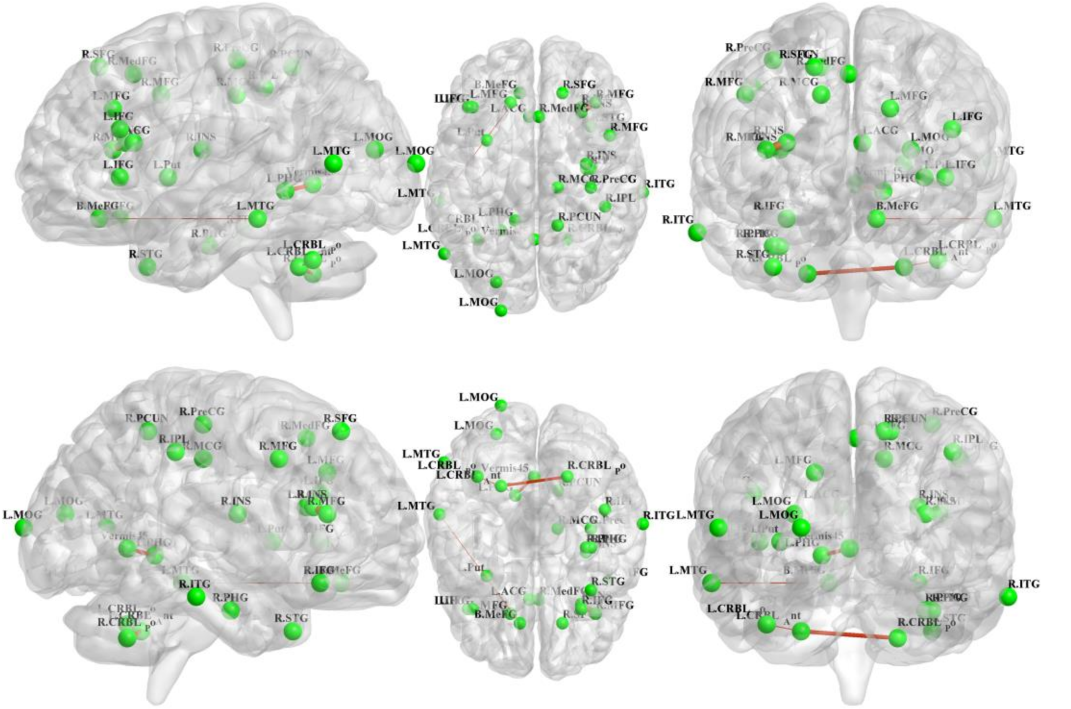

(3) aMCI group r = 0.5

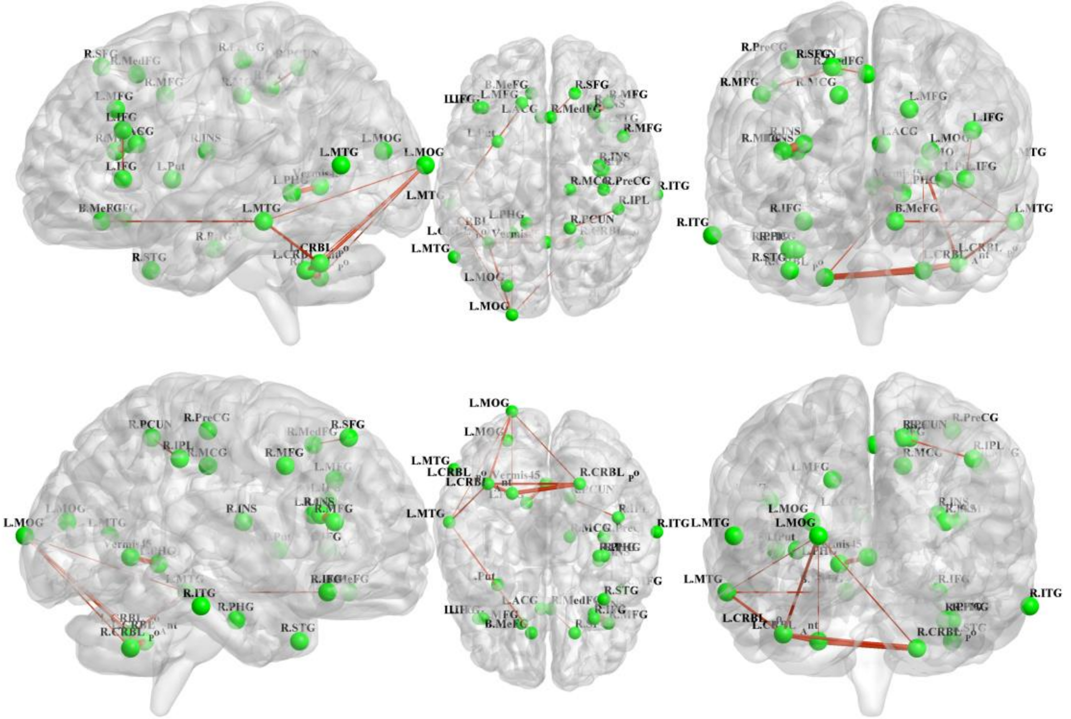

(4) aMCI group r = 0.7

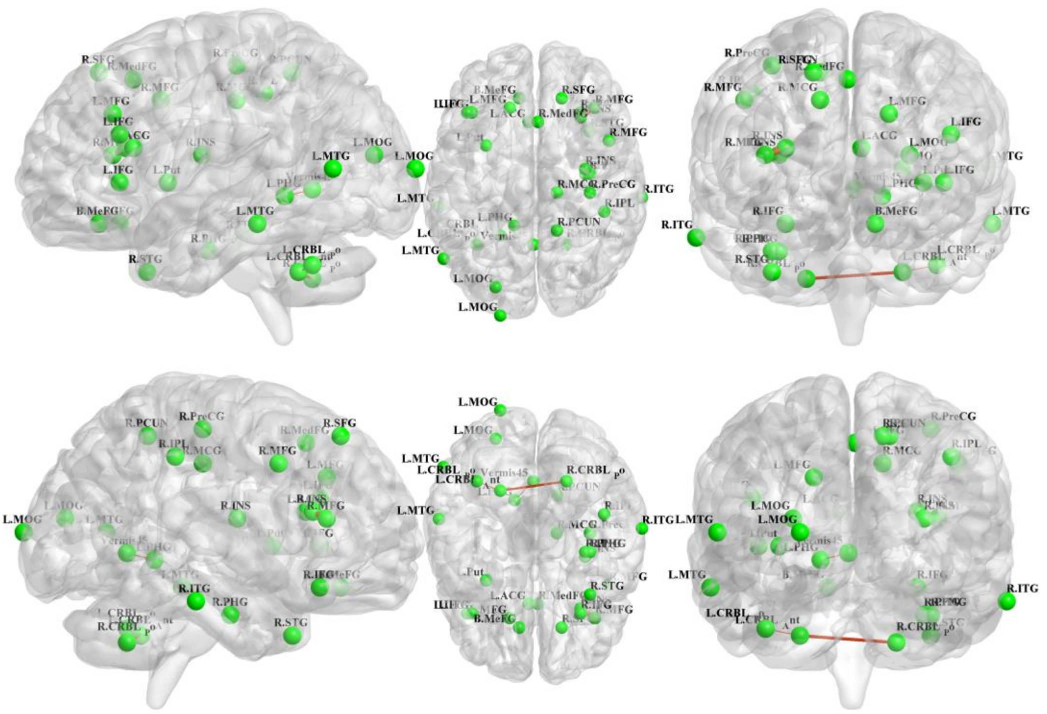

Part IV. Differences between groups in the functional connectivity of unidirectional weighted network at thresholds of $P < 0.05$, 0.01 and 0.001, respectively.

(1) $P < 0.05$

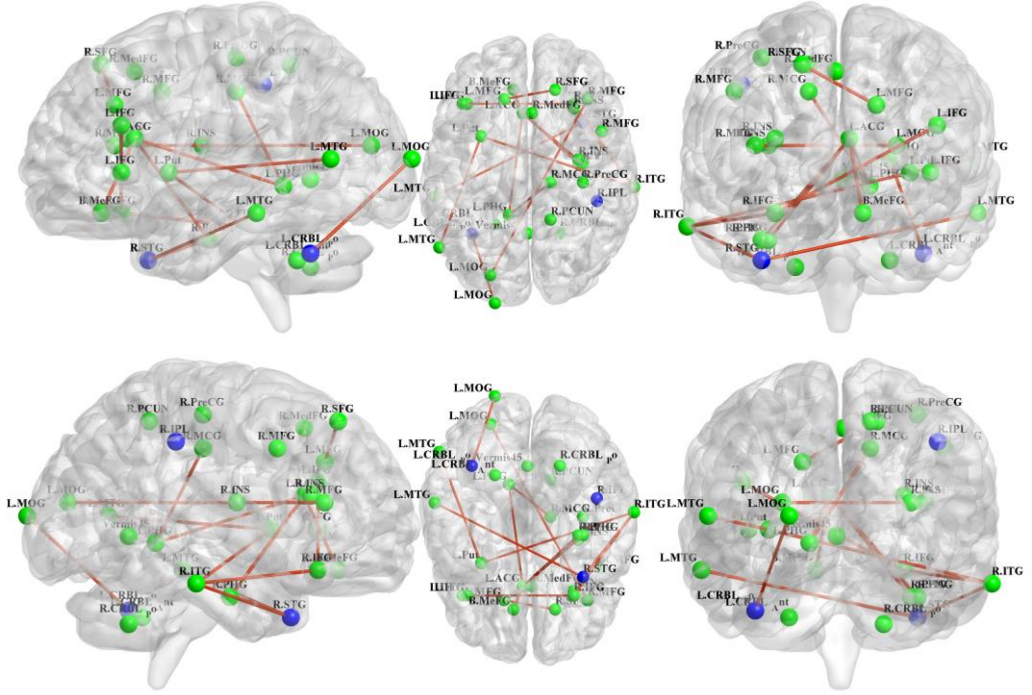

(2) $P < 0.01$

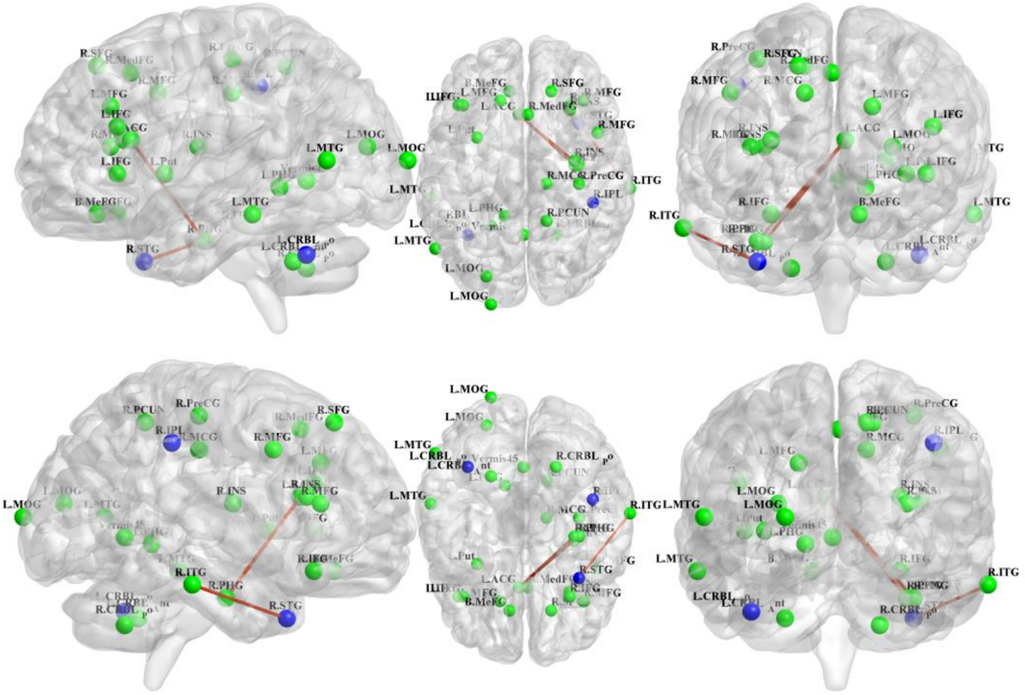

(3) $P < 0.001$

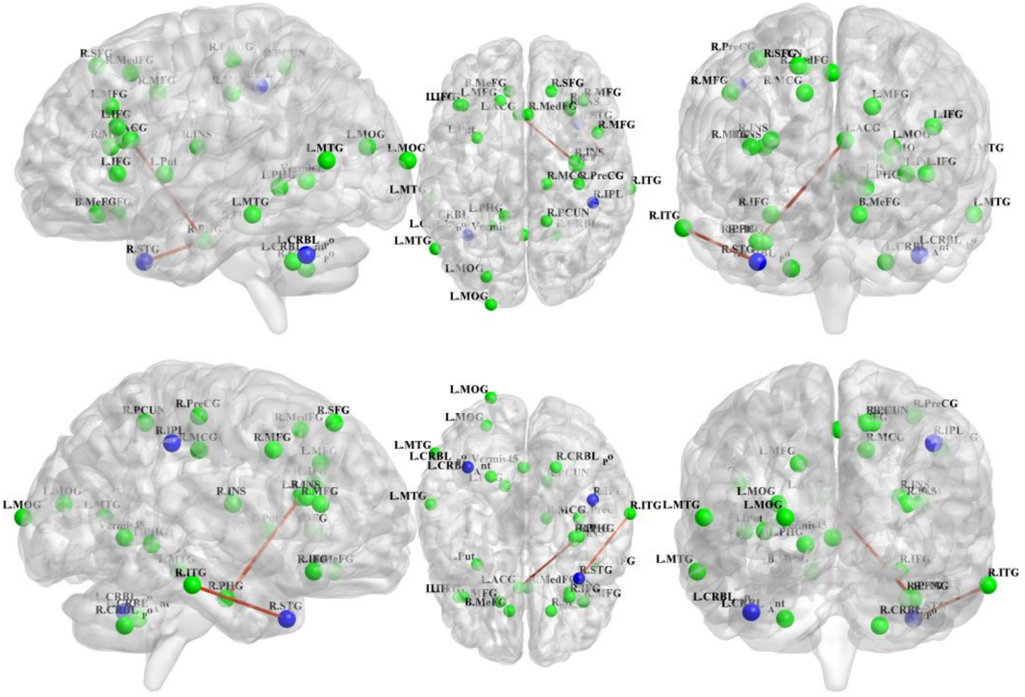

Supplement: Supplementary file 1 [file oncotarget-07-15315-s001.pdf]
